# ATTACHMENT SECURITY AND VIEWS TOWARD AGING IN OLDER COUPLES: A DYADIC PERSPECTIVE

**DOI:** 10.1093/geroni/igz038.319

**Published:** 2019-11-08

**Authors:** E-Shien Chang, Seline O Goktas, Joan K Monin

**Affiliations:** 1 Yale School of Public Health, New Haven, Connecticut, United States; 2 Yale School of Medicine, New Haven, Connecticut, United States

## Abstract

Research has shown that attachment security–feelings of emotional safety from interpersonal closeness and responsiveness– is associated with better appraisal of stressful situations. Individuals’ views toward aging could be one avenue such appraisals are expressed that in turn contribute to better health in late life. However, no studies to our knowledge have examined the dyadic associations between attachment security and views towards aging in the context of close relationships. We hypothesized that attachment insecurity would be associated with individuals’ own and partners’ negative views toward aging in older married couples. The study sample was comprised of 77 older persons with a self-reported musculoskeletal condition and their caregiving spouses. The Experiences in Close Relationships Scale and the open-ended Image of Aging questions were used to measure attachment security and views toward aging. Data were analyzed with SPPS mixed models using the Actor Partner Interdependence Model. Mean age of care-recipients were 65.9 and 64.8 for their spouses. Contrary to our hypothesis, results showed no significant associations between each individual’s attachment security and their own views toward aging. However, care-recipients reported particularly positive views toward aging when caregivers had low attachment anxiety (p=.03), and caregivers reported more negative views toward aging when care-recipients had low attachment avoidance (p=.02). Findings suggest that having a close partner who is securely attached may be protective of one’s own views of aging, which may in turn have positive effects on health.

